# Ionization Gas Sensor Using Suspended Carbon Nanotube Beams

**DOI:** 10.3390/s20061660

**Published:** 2020-03-17

**Authors:** Shivaram Arunachalam, Ricardo Izquierdo, Frederic Nabki

**Affiliations:** Department of Electrical Engineering, École de Technologie Supérieure, Montreal, QC H3C 1K3, Canada; ricardo.izquierdo@etsmtl.ca (R.I.); frederic.nabki@etsmtl.ca (F.N.)

**Keywords:** suspended, carbon nanotubes, ionization

## Abstract

An ionization sensor based on suspended carbon nanotubes (CNTs) was presented. A suspended CNT beam was fabricated by a low-temperature surface micromachining process using SU8 photoresist as the sacrificial layer. Application of a bias to the CNT beam generated very high non-linear electric fields near the tips of individual CNTs sufficient to ionize target gas molecules and initiate a breakdown current. The sensing mechanism of the CNT ionization sensor was discussed. The sensor response was tested in air, nitrogen, argon, and helium ambients. Each gas demonstrated a unique breakdown signature. Further, the sensor was tested with gaseous mixtures. The sensor exhibited good long-term stability and had comparable performance to other reported CNT-based ionization sensors in literature, which use high-temperature vapor deposition methods to grow CNTs. The sensor notably allowed for lower ionization voltages due to its reduced ionization gap size.

## 1. Introduction

Carbon nanotube (CNT)-based gas sensors have been the subject of much research in the past few decades owing to their excellent electrical and mechanical properties. A host of sensing principles [1,2,3,4] and device designs have been proposed to develop gas sensors based on CNTs with high-performance metrics [5]. Other materials that have been used in gas sensing applications include metal oxides [6,7] and polymers [8,9]. Although every gas-sensing material possesses their advantages, they are also associated with certain drawbacks, such as cross-sensitivity, performance degradation over time, and shelf-life. CNT-based sensors have overcome these drawbacks and have proven to be excellent gas sensing elements [10]. Suspended chemical CNT chemical gas sensors have also been previously fabricated [11,12]. Chikkadi et al. fabricated a NO_2_ sensor using a single suspended CNT across prefabricated electrodes. Li et al. [13] fabricated suspended CNT microlines on a polymeric substrate using a wet contact printing method. More recently, suspended chemical CNT sensors have been used to sense humidity [14] with minimal hysteretic effects due to the mitigation of substrate effects [15]. The increase in surface area for adsorption of the analyte also results in better sensitivity and long-term stability of the sensor. However, most chemical sensors are still prone to chemical poisoning and cross-sensitivity to other gases.

Ionization gas sensors operate by detecting gases based on their ionization properties and the field emission properties of the sensing elements. Townsend first studied and postulated the concept of gaseous breakdown, thereby formulating the understanding of this sensing principle’s physics and its operation [16]. Ionization occurs due to the migration of electrons from the cathode to anode under an applied electric field between two electrodes. The main advantage of ionization gas sensors is their independence on absorption characteristics of the sensing material or chemical interaction between species. Thus, they have faster response and recovery time and are also sensitive to inert gases. Some drawbacks of ionization sensors include relatively high voltage operation, bulky architecture, and performance degradation over time. Metal oxides, ceramics, and, more recently, carbon-based materials have been used to fabricate ionization gas sensors. CNT-based ionization sensors work on the principle of the formation of a corona on the tips of the individual CNTs due to very high electric fields near the tips that favor discharges at lower voltages than that of typical ionization sensors. On the contrary, typical ionization sensors based on metal electrodes suffer from high power consumption and high-temperature operation [17,18,19]. A CNT-based ionization sensor was proposed in 2003 by [20] using vertically aligned multi-walled CNTs. The sensor has shown good sensitivity to various gases. A comparative study showed that the breakdown voltages for CNT-based sensors are much lower than a sensor with metal electrodes. However, no information is provided about the long-term stability of the sensor, which is a very important performance metric of gas sensors. Other CNT-based ionization sensors have been developed to detect humidity, NO_2_, etc. [21,22,23,24]. In all the articles mentioned above, the CNTs are grown vertically using very high temperature-based vapor deposition techniques. These growth techniques have a risk of low yield. Moreover, these deposition techniques are not suited to the integration of the sensor with a wide range of substrates, which can reduce their degree of integration in a full implementation. 

In this work, we presented an ionization gas sensor based on suspended single-walled CNTs that were arranged horizontally, instead of being vertically aligned like previously mentioned publications. The resultant sensor showed similar behavior to vertically aligned CNT ionization sensors. It is worthwhile to note that the fabrication process had a temperature budget that was below 115 °C and, as such, had an advantage if the sensor was to be integrated with a wide range of substrates or various metallizations.

## 2. Materials and Methods

**Figure 1:**
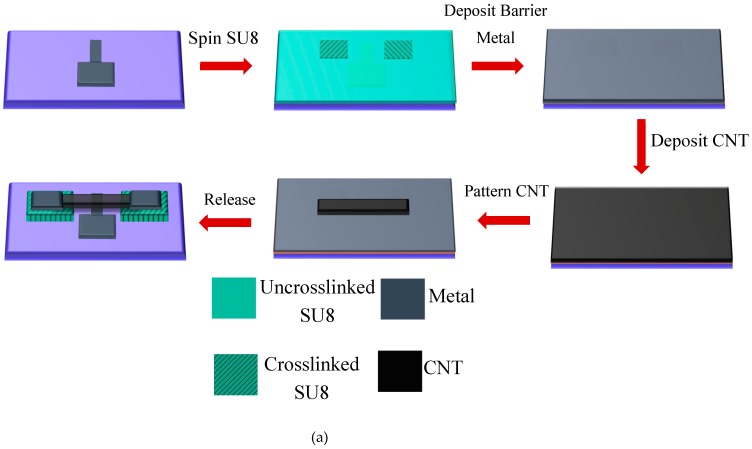

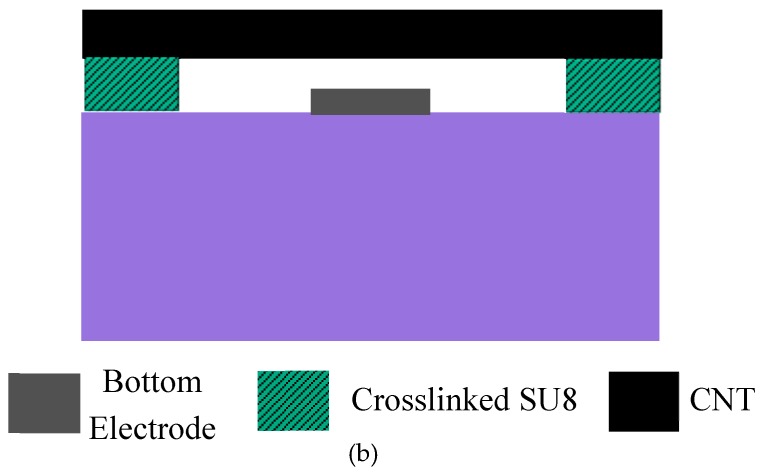
(**a**) Schematic of thefabrication process. Reprinted with permission from [25], and (**b**) Schematic of the cross section of the device.

The fabrication process used uncrosslinked SU8 photoresist as a sacrificial layer. The process was originally developed to obtain suspended nanotube beams and is shown in Figure 1a, and a cross-section of the device is shown in Figure 1b. To begin, a 30 nm-thick aluminum layer was deposited onto a silicon substrate by thermal evaporation using a Joule evaporator and patterned into an electrode shape using contact UV lithography and wet etching, using an Aluminum Etchant (Sigma Aldrich, St. Louis, Missouri, USA) etching solution.

Then, a 3.6 µm-thick SU8 2010 photoresist, obtained from Microchem (Westborough, Massacheusetts, USA) and diluted with cyclopentanone in the ratio 1:4, was spun on top of the metal electrode and partially cross-linked by UV contact lithography using an OAI aligner to form pillar anchors for the CNT beam. The dilution was necessary to achieve the desired thickness using the thicker SU8 2010 resist. Following this step, a 30 nm-thick barrier aluminum metal layer (Al) was deposited over the SU8 by thermal evaporation using a Joule effect evaporator.

This barrier metal helped prevent the dissolution of the SU8 in future processing steps, more specifically during the CNT film transfer onto the substrate. The CNTs used in this work were obtained from Carbon Solutions Inc. (Riverside, California, USA) (P2-SWNT), and a solution of CNTs in water was prepared by dispersing CNTs in a 1 wt% solution of sodium dodecyl sulfate (SDDS) (obtained from Sigma Aldrich St. Louis, Missouri, USA) in water and ultrasonicated for 6 hours followed by centrifugation for 60 minutes followed by decantation. Then, a thin film of CNTs was prepared by vacuum filtration with a thickness of 0.48 µm verified by profilometry. The reason for using the vacuum filtration technique is the easiness of processing and the ability to control the film thickness precisely depending on the amount of solution filtered. The film was then washed with deionized water, air-dried, and transferred onto the substrate. The CNT beams were patterned by UV lithography followed by oxygen plasma ashing. A 20 nm-thick aluminum film was deposited, again by thermal evaporation and contacts, was then patterned via lithography and wet etching onto the beam’s extremities, above the anchors.

**Figure 2:**
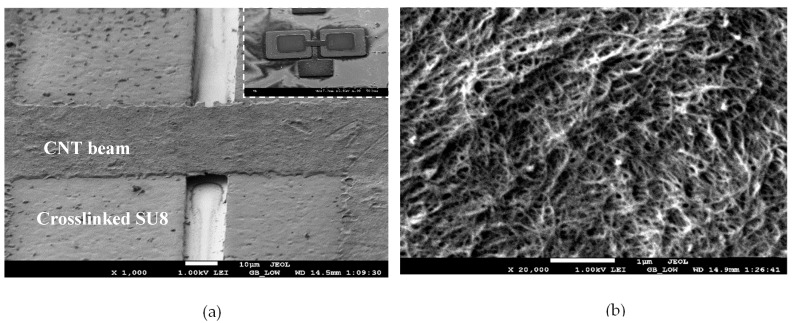
(**a**) SEM micrograph of the device. The inset in the figure shows a top view of the device structure with the bottom electrode, and (**b**) High magnification SEM micrograph showing a “mat” of carbon nanotubes (CNTs) comprising the beam. Reprinted with permission from [25].

Finally, the barrier metal was etched using wet etching, and this was followed by removal of the uncrosslinked SU8 in RemoverPG obtained from Microchem (Westborough, Massacheusetts, USA) to release the CNT beams. This yielded a gap between the suspended CNT beam and the bottom electrode of 3.6 μm. All the processing steps were below a temperature budget of 115 °C, making the process amenable to monolithic integration with electronics or other sensors. This fabrication process had been adopted from [25] and was derived from the process in [14] used to fabricate CNT humidity sensors. However, the fabrication process diverged from the work in [14] by adding the bottom electrode layer to implement the ionization sensor. The SEM micrograph of the fabricated device is shown in Figure 2a. The inset of the figure shows the overall device structure with the bottom electrode and the CNT beam suspended above it. Figure 2b shows a high magnification SEM image of the CNT networks that comprise the overall beam structure. Evidently, the SEM micrograph in Figure 2b shows the intertwined CNTs that form a network aiding in the ionization process.

### Test Setup

**Figure 3:**
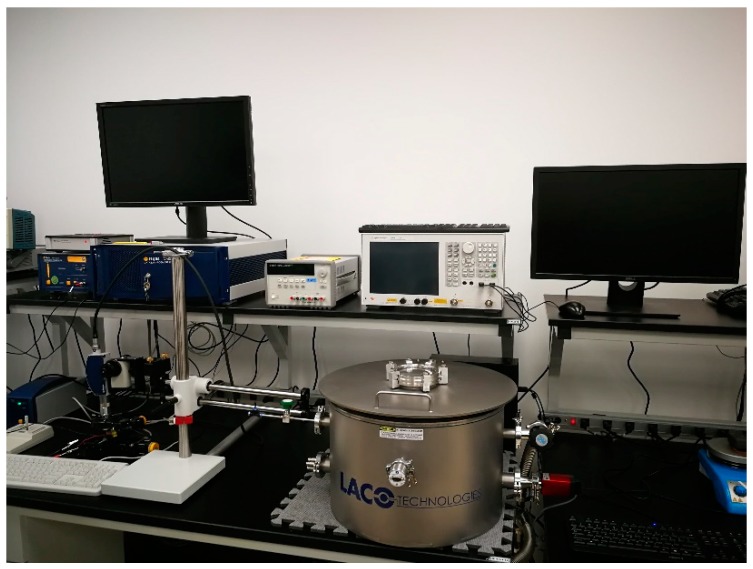
Photograph of the test setup used for the measurements showing the gas chamber, the multimeter, and the computer for data acquisition.

Figure 3 shows the photograph of the test setup. The setup consisted of a custom gas chamber obtained from Laco Technologies(Salt Lake city, Utah, USA) in which the device was placed. Two DC probes were placed within the chamber along with micro-positioners to align the probes. The chamber was evacuated using a vacuum pump. Gas lines were connected to the chamber in order to inject different gases for sensor characterization. The gas flow was controlled via mass flow controllers. The DC probes were in-turn connected to a Keithley 2290-5 series high voltage DC source limited to 5000 V in order to extract the ionization voltage. The concentration of the gas inside the chamber was measured using a commercial sensor embedded within the chamber.

## 3. Results

**Figure 4:**
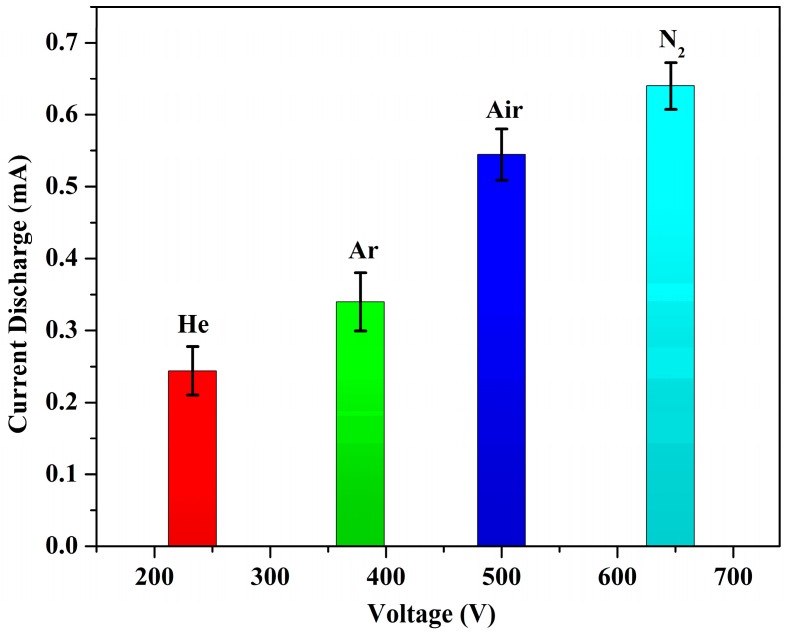
Breakdown signatures of various gases as tested. Reprinted with permission from [25]. The trend of breakdown voltages was similar to that observed by Modi et al.

A plot of the breakdown voltages of each tested gas as a function of the discharge current is shown in Figure 4. For six measured devices, the average breakdown in the air was observed at a voltage of 500 V with a discharge current of 0.44 mA. In a vacuum, there was no observed breakdown for a voltage sweep between 150 V and 1000 V. This was expected since, in a vacuum, there are not enough gaseous molecules in the chamber for ionization to occur.

**Figure 5:**
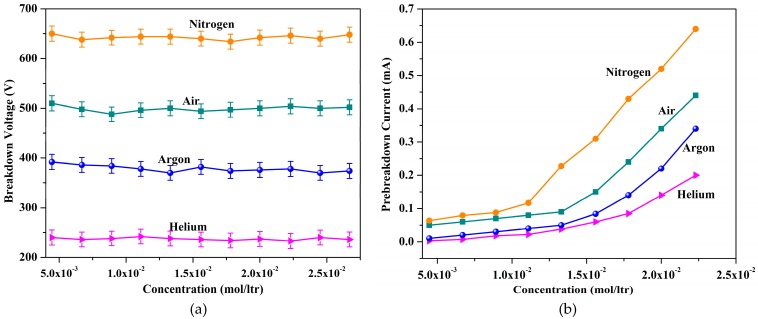
(**a**) Gas concentration vs. breakdown voltage, which shows that the breakdown voltage of the gas was independent of the gas concentration, and (**b**) discharge current increased as a function of concentration due to increasing gas molecules available for conduction.

Hence, different gases showed breakdown at different voltages. The effect of gas concentration on the breakdown voltages of each gas is shown in Figure 5a. The breakdown voltages did not change significantly with varying the concentration. This was because the breakdown behavior was dominated by the generation of highly non-linear electric fields at the tips of the CNTs. The electric field bridged the inter-electrode gap, thereby mitigating the effect of gas concentration on the breakdown voltage.

**Figure 6:**
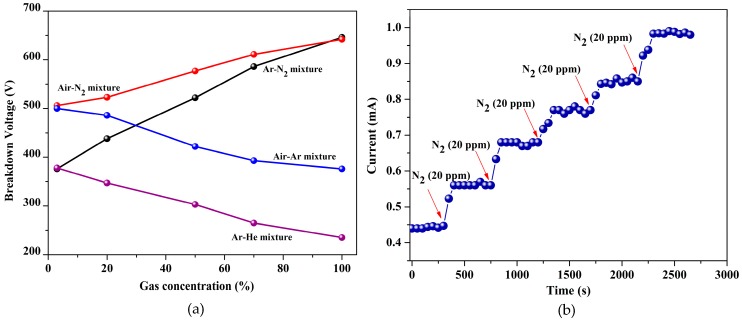
(**a**) Breakdown voltage variation with different gas-air mixtures. The breakdown voltage increased with increasing N_2_ concentration and decreased with increasing Ar concentration, and (**b**) Dynamic sensing curves of the sensor for the air-N_2_ mixture_._ The breakdown current increased as the relative concentration of N_2_ increased in steps of 20 ppm.

Figure 5b shows the discharge current as a function of gas concentration. The discharge current increased logarithmically as the concentration increased, which was because the generated current at the breakdown was a property related to the number of gas molecules amenable to conduction at the time of the breakdown. Thus, the self-sustained discharge could also be used to quantify the gas concentration. The device was also tested for sensitivity to gaseous mixtures, as shown in Figure 6a. Here, the gas concentration percentage outlined the mixture ratio of the second gas listed in each mixture to the overall mixture. In the first case, argon was pumped into the chamber, already containing air. For greater than 50% argon concentration in the chamber, the ionization voltage converged to the value obtained for standalone argon measurements indicated the increased presence of argon gas in the chamber. As the relative concentration of argon was reduced, the voltage increased towards 500 V, a value characteristic of the ionization voltage measured for air. Then, the air-argon mixture was pumped out, and a mixture of air-nitrogen was pumped into the chamber. As the amount of N_2_ was increased, the voltage increased towards the breakdown voltage of 646 V for nitrogen. As the concentration of nitrogen was decreased gradually, the breakdown voltage also decreased. Similarly, the sensor was also tested in other gaseous mixtures, such as argon-helium. In this case, the recorded voltage shifted towards 378 V with decreasing helium concentration and towards the standalone value of 233 V as the helium concentration increased, as predicted. Figure 6b shows the dynamic sensing curve of the sensor for the air-nitrogen mixture. The current increased from 0.44 mA in air ambient to 0.93 mA for 100 ppm of N_2_.

Then, when nitrogen was introduced into the chamber, the average breakdown was observed at 646 V, with a discharge current of 0.618 mA. In argon ambient, the average breakdown was observed at 378 V, with a discharge current of 0.32 mA, and, for helium, the average breakdown was 233 V. The measurements were made multiple times in order to verify the stability of the breakdown voltages and to determine the repeatability of the sensor. The trend of the breakdown voltage of the tested gases is similar to other reports in the literature [20]. This corresponds to the theory of different gases having different breakdown “signatures”. The ionization of different gases occurs via a Townsend discharge where there is an electron avalanche due to the ionization. The avalanche effect depends on the mean free path of gas molecules, which varies depending on the gas density.

**Figure 7:**
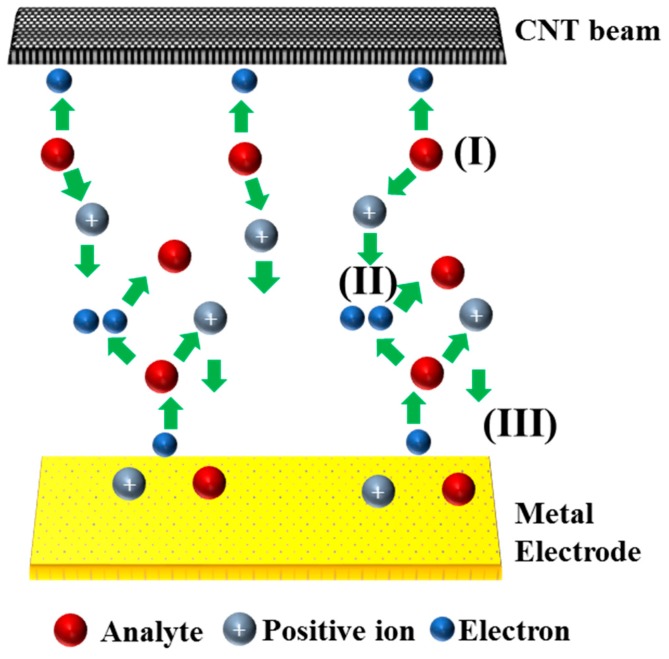
Sensing mechanism with a positive nanotip: (I) Nanotip Ionization, (II) Impact Ionization, and (III) Electron Recombination.

**Figure 8:**
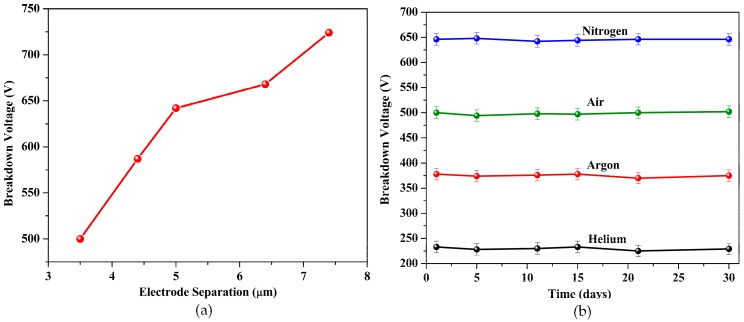
(**a**) Effect of inter-electrode distance on the breakdown voltage, and (**b**) Long-term stability of the sensor with tests performed at intervals of 5 days. The breakdown voltage did not vary significantly with time.

The sensitivity of the sensor defined as the ratio of the gas concentration to changes in the current and is given by:

*S* = *C*/(*I_g_* − *I_a_*) (1)

where *S* is the sensitivity, *C* is the concentration, *I_g_* is the current in the gas ambient, and *I_a_* is the pre-breakdown current in the air. The sensitivity of the sensor was about 0.2 ppm/0.01 mA. Thus, the limit detection of the sensor was about 0.2 ppm. The schematic of Figure 7 shows the possible sensing mechanism with a positive CNT nanotip. The tips of the CNTs produced strong electric fields, which ionized the target gas analyte and resulted in the release of an electron. The released electron was absorbed at the positive CNT tip, while the ion moved towards the negative electrode (metal). On the metal surface, the ion recombined with an electron. Further, the kinetic energy of the ion induced an electron emission from the negative electrode (also called the γ process). The emitted electron now had enough energy to ionize other gas molecules on its path. This process is called impact ionization. The pre-breakdown current was due to the ionization at the nanotip, which was amplified by the impact ionization. Thus, the working voltage of the device could be reduced [26]. When the CNTs were biased as the negative electrode, electron emission occurred from the CNT nanotips, followed by impact ionization and recombination, which would further aid in decreasing the working voltage of the device. When the CNTs were biased negatively, the breakdown voltage in the air reduced to 242 V, while, for nitrogen, the breakdown was observed at 328 V, in agreement with [26] and [27]. The one-dimensional structure of CNTs aided in amplifying the electric field and induce discharge at a lower voltage as compared to traditional metallic ionization sensors. This was in accordance with [20], where the use of two metallic electrodes in the air for ionization yielded breakdown at 1050 V as compared to 354 V for CNTs for similar inter-electrode separation. Figure 8a shows the effect of interelectrode separation on the breakdown voltage. The different electrode spacing was achieved by varying the SU8 pillar thickness during the fabrication process. The breakdown voltage for the gases was found to decrease with the reduction in the inter-electrode spacing. For air, the breakdown voltage was observed at 725 V for the spacing of 7.4 µm and decreased to 500 V for the spacing of 3.6 µm. The breakdown voltage could then further be reduced by decreasing the inter-electrode distance. In this case, the limitation was the beam collapse onto the bottom electrode below a height of 3.6 µm, possibly due to stiction issues.

The inter-electrode distance could also be decreased by using thinner resists as sacrificial materials, and this is currently being investigated with thinner SU8 variants. The reason for using SU8 as the sacrificial material was its ease of availability and its ease of processing. Further, SU8 also allowed for a higher thermal budget and provided a stable surface for chemical processing. The long-term stability of the sensor was gauged by measuring the breakdown voltage of all gases after intervals of 5 days for a period of 30 days. The sensor exhibited stable and consistent behavior for each measurement phase for all tested gases, as shown in Figure 8b. The sensor was also tested for performance degradation under a constant applied voltage of 500 V. The sensor showed consistent behavior for 150 breakdown cycles at 500 V, after which the CNT network seemed to degrade. It remains to be seen if increasing the thickness of the CNT layer influences the degradation time of the sensor.

## 4. Discussion

The work presented here shows the application of suspended CNTs to ionization gas sensing. In comparison to metallic ionization gas sensors, CNTs offer advantages, such as room temperature operation, reduced operating voltages, and miniature size. Ionization sensors can be advantageous in applications, which require instant and consistent gas detection. The generation of strong non-linear electric fields at the tips of CNTs under applied bias reduces the breakdown voltages of target gases in comparison to metallic ionization sensors. Under positive bias, the sensing proceeds via three different steps, namely, the ionization of the CNT tips, the ionization of the gas due to impact with the electron, and the recombination. It is important to acknowledge that the operation of the presented ionization sensor is not readily applicable in all sensing systems due to the still high breakdown voltages required. However, in comparison to other CNT-based ionization sensors [20,22,23], the presented suspended CNT sensor demonstrates lower breakdown voltages and represents a step towards mitigating this limitation. Methods to reduce the breakdown voltage even further are being investigated to widen the applications of the sensor. The sensor also shows predictable behavior on exposure to gaseous mixtures. The analyte concentration can be quantified by monitoring the breakdown current. Ionization sensors could be used in place of gas chromatography systems, which are often bulky and expensive, provided the working voltage is of the order of a few volts. The high working voltage of ionization sensors hinders them from being used as-is in such an application. Further, sensor decay also remains an issue to be studied. It could be interesting to see if the sensor can also be used to sense volatile organic compounds (VOCs), thereby providing an alternative for gas chromatography systems. Further, the operation of the sensor in various temperature ranges and humidity needs to be studied.

## 5. Conclusions

In this work, an ionization gas sensor based on suspended CNTs was demonstrated as an alternative to conventional CNT ionization sensors. A metal electrode was placed under the suspended beam and was used as one electrode with the suspended beam acting as the other electrode. The CNT beam was biased both positively and negatively, and the sensor was able to sense various inorganic gases based on their breakdown voltages. In comparison to metallic ionization sensors, the use of CNTs lowered the working voltage of the sensor, charting a path towards a more compact and wider application space for such sensors. The demonstrated sensor could be used for applications, such as environmental monitoring and gas sensing, in industrial and domestic environments.
